# Leptospirosis vaccines

**DOI:** 10.1186/1475-2859-6-39

**Published:** 2007-12-11

**Authors:** Zhijun Wang, Li Jin, Alicja Węgrzyn

**Affiliations:** 1 CAS-MPG Partner Institute for Computational Biology, Shanghai Institutes for Biological Sciences, Chinese Academy of Sciences, 200031, Shanghai, PR China; 2 Key Laboratory of Medical Molecular Virology, Shanghai Medical College, Fudan University, 200032, Shanghai, PR China; 3 MOE Key Laboratory of Contemporary Anthropology and Center for Evolutionary Biology, School of Life Sciences and Institutes of Biomedical Sciences, Fudan University, 200433, Shanghai, PR China; 4 Laboratory of Molecular Biology (affiliated with the University of Gdańsk), Institute of Biochemistry and Biophysics, Polish Academy of Sciences, 80-822 Gdańsk, Poland

## Abstract

Leptospirosis is a serious infection disease caused by pathogenic strains of the *Leptospira *spirochetes, which affects not only humans but also animals. It has long been expected to find an effective vaccine to prevent leptospirosis through immunization of high risk humans or animals. Although some leptospirosis vaccines have been obtained, the vaccination is relatively unsuccessful in clinical application despite decades of research and millions of dollars spent. In this review, the recent advancements of recombinant outer membrane protein (OMP) vaccines, lipopolysaccharide (LPS) vaccines, inactivated vaccines, attenuated vaccines and DNA vaccines against leptospirosis are reviewed. A comparison of these vaccines may lead to development of new potential methods to combat leptospirosis and facilitate the leptospirosis vaccine research. Moreover, a vaccine ontology database was built for the scientists working on the leptospirosis vaccines as a starting tool.

## 1. Background

**Figure 1:**
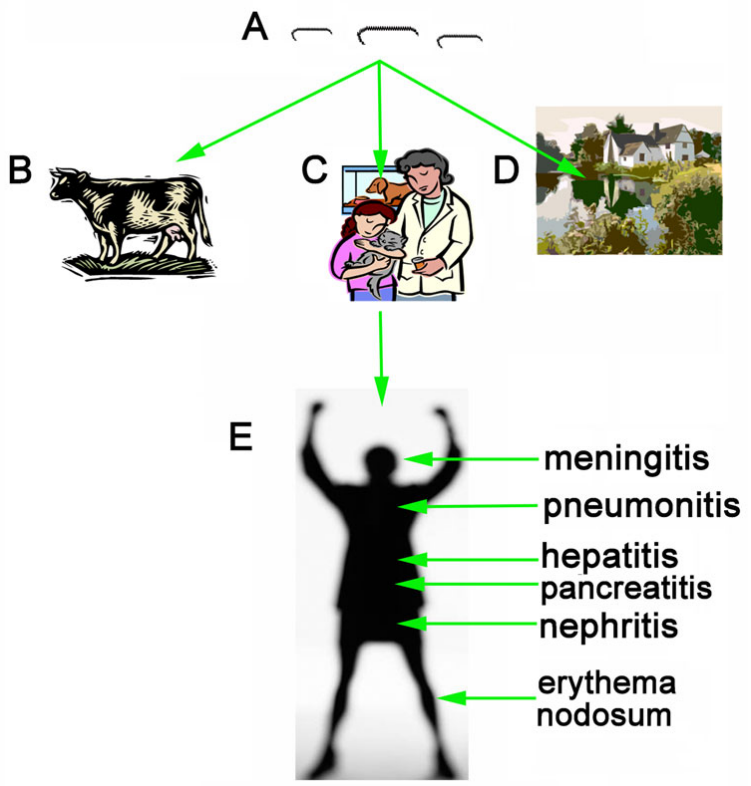
The infection pathway of Leptospire. A): Leptospires in the nature resource. B): Leptospires in the rodents or wield animals. C): Leptospires in pets. D): Leptospires in water or soil. E): The infected human can develop meningitis, pneumonitis, hepatitis, pancreatitis, nephritis and erythema nodosum. The organisms are excreted in urine. They survive for longer periods in natural waters.

**Figure 2:**
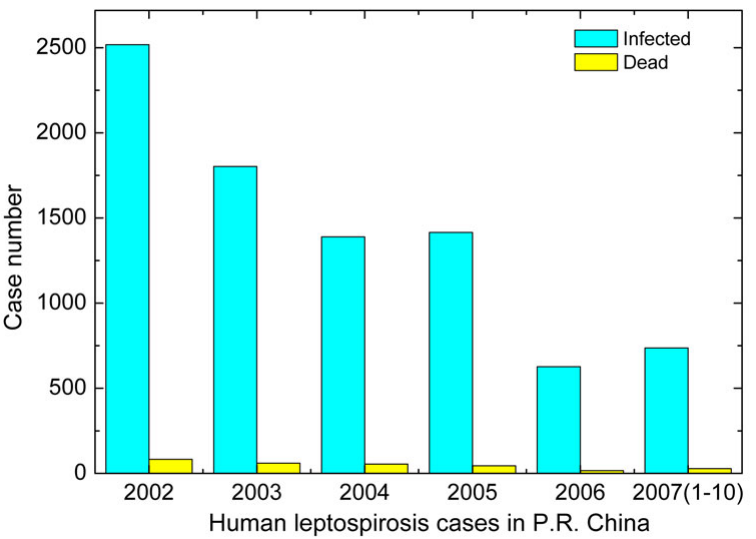
Human leptospirosis cases in People's Republic of China during 2002–2007 in China mainland. The data were reported by the Ministry of Health of the People's Republic of China. The blue bars are the infected cases, and the yellow bars are the dead cases. The data of 2007 are the infected and dead human leptospirosis cases from January to October.

Leptospirosis is a widespread disease [1], caused by infection with the spirochete bacterium *Leptospira*, which affects almost all mammals [1-13]. Leptospirosis was initially described as Weil's syndrome [8,14]. It is predominantly an occupational disease which affects humans who come into frequently contact with rodents, pets or polluted water [15-18] (Fig. 1). Infection is facilitated with penetrating leptospires through mucosa or an open skin [19]. After gaining entry through the skin, the bacterium causes a serious disease [19]. The symptoms of leptospirosis are extremely broad from meningitis [20], pneumonitis [21,22], hepatitis [23], nephritis [24-27], pancreatitis [28] and erythema nodosum [29] and death [30,31]. Fig. 2 shows the data of human leptospirosis cases reported by Ministry of Health of the People's Republic of China from January 2002 to October 2007 in China mainland. During this time, about 1,500 infected cases and 50 dead were reported. However, many human leptospirosis cases might be misdiagnosed or omitted due to poor medical care and information. *Leptospira *has over 200 pathogenic serovars, and divides into 25 serogroups, and many different strains with small antigenic differences can be found in some serovars [2,17].

Leptospires have evolved ways to escape the immune defense. Pathogenic leptospires are able to translocate through cell monolayers at a rate significantly greater than that of nonpathogenic leptospires [32]. The rapid translocation of pathogenic leptospires between mammalian cells allows the bacteria to quickly reach the bloodstream and disseminate to multiple organs [32]. Virulent leptospires can rapidly enter kidney fibroblasts and induce a programmed cell death [33]. Thus, it is a challenge for immunologists to develop an effective and safe leptospirosis vaccine [34-37]. Currently, molecular and cellular studies on leptospirosis vaccines have been focused on bacterial motility [38,39], lipopolysaccharides (LPSs) [10,40-47], lipoproteins [48-56], outer-membrane proteins (OMPs) [52,53,57-62] and potential virulence factors [39,63-68]. However, it is still a lack of an extensive knowledge-based annotation of leptospirosis vaccines for the scientists working in the field of leptospirosis vaccines. It has inspired us to investigate the current developments of leptospirosis vaccines and to construct a database of the leptospirosis vaccines for incorporating the leptospirosis vaccine information into bioinformatics by using Microsoft SQL server technology and intelligent algorithms (see section 6 and website [69]).

Here, we classified the leptospirosis vaccines into recombinant protein vaccines, lipopolysaccharide (LPS) vaccines, inactivated and attenuated vaccines, and DNA vaccines for reviewing the current advancements in leptospirosis vaccine research.

## 2. Recombinant protein vaccines

Recombinant protein vaccines have a great potential against leptospirosis. Several leptospiral recombinant protein vaccines have been constructed with modern biotechnological methods [48,53,56,58,61,70-75], of which recombinant OMPs, lipoproteins, and virulence factors acquired a considerable interest.

### 2.1. Recombinant OMP vaccines

The protective characteristics of several recombinant OMP vaccines have been tested, including leptospiral outer membrane protein OmpL1, lipoprotein LipL41 [53], hemolysis-associated protein 1 (Hap1) [76] and immunoglobulin-like (Lig) protein [77]. In 1993, the first leptospiral OMP protein, a 31 kDa surface protein OmpL1 of *Leptospira*, was reported [78]. 6 years later, studies on the Golden Syrian hamster model of leptospirosis demonstrated immunoprotective effects of the leptospiral outer membrane protein OmpL1 and lipoprotein LipL41 [53]. However, after another 2 years, it was reported that adenovirus-mediated OmpL1 failed to protect gerbils against heterologous *Leptospira *infection, contrary to adenovirus-mediated Hap1, which induced a significant protection [76]. In 2002, a 130 kDa immunoreactive leptospiral immunoglobulin-like protein A (LigA) from *L. interrogans *was described [77]. Then, the immune response of LigA proteins was confirmed, as two immunoglobulin-like proteins, LigA and LigB, induced a protection against leptospires [56,73,74]. These results indicated that LigA and LigB may play an important role in the host cell attachment, as well as invasion during leptospiral pathogenesis [79-81]. Moreover, the *lig *gene was shown to be useful in the detection of pathogenic *Leptospira *[82].

Several leptospiral outer membrane proteins, e.g. LAg42, Loa22, Lk73.5, have been recognized as leptospirosis vaccine candidates, but they were not tested in animal models for vaccine development. LAg42 is a 42 kDa inner-membrane protein. It was identified in pathogenic *Leptospira *as a factor involved in virulence [83]. Loa22 was found among pathogenic leptospires but not in non-pathogenic leptospires. It is located in the outer membrane and exposed on the cell surface. It has been considered as a candidate for a novel vaccine against leptospirosis [49]. Lk73.5 is a host-inducible immunogenicity protein from pathogenic *L. interrogans *[72]. Although the protective characteristics of LAg42, Loa22 and Lk73.5 are not available, we believe these outer membrane proteins might be suitable as vaccine candidates.

### 2.2. Recombinant lipoprotein vaccines

Lipoproteins are important proteins in leptospires. These proteins are abundant in the outer membrane, to which they are attached through fatty acids. Because of difficulty in production of lipoproteins in heterologous expression systems, only LipL41 was reported as a potential vaccine. However, many lipoproteins (for example: LipL32, LipL45 and LipL21) could be suitable as vaccine candidates. LipL32 [52] and LipL41 were identified as targets during natural infection by leptospires [37]. They are potentially useful for serodiagnosis and may serve as targets for vaccine design. LipL45 [67] and LipL21 [50] were described as surface membrane lipoproteins that are produced during infection and conserved among pathogenic *Leptospira *species. LipL45 is produced as a 45-kDa lipoprotein and it is processed to a 31-kDa C-terminal form, P31_LipL45 _[67]. LipL45 is also called Qlp42 [84].

Moreover, the lipoprotein-like complex glycolipoproteins (GLPs) were suggested as vaccine candidates. A glycolipoprotein (GLP) extracted from either pathogenic *L. interrogans *or nonpathogenic *L. biflexa *was shown to induce production of the tumor necrosis factor, interleukin-10 and CD69 [85]. Obviously, the reported lipoproteins (LipL32, LipL45, LipL21 and GLP) are leptospirosis vaccine candidates.

On the other hand, lipoprotein LipL36 was found not suitable as leptospirosis vaccine. LipL36 is a 36 kDa leptospiral outer membrane lipoprotein [54], which is synthesized at 30°C, but not at 37°C *in vivo *[84,86]. Production of this protein was downregulated in host-adapted leptospires, suggesting that it is not involved in pathogenesis after entry into the mammalian host [62].

### 2.3. Recombinant virulence factor vaccines

Only a few papers were reported to identify leptospiral virulence factors, including FlaA, FlaB [87], Hsp58 [88], SphH [89,90] and ChpK [91]. FlaA and FlaB are important components of leptospiral periplasmic flagella (PF). PF is a complex structure, composed of a core, surrounded by two sheath layers, which are important virulence factors of *Leptospira *[92]. In most spirochete species, the core of PF consists of at least three proteins: FlaB1, FlaB2 and FlaB3. The FlaA protein forms a sheath around the FlaB core. FlaA, together with FlaB, impact PF helical morphology [87].

Using *flaA::cat, flaA::kan, flaB1::kan, flaB2::cat *and *flaB3::cat *mutants, it was shown that these strains were less motile than the wild-type strain [38]. These results indicate that FlaA and FlaB are virulence factors of *Leptospira*. The gene encoding the FlaB virulence factor, *flaB*, can be amplified from the genomic DNA of several pathogenic serovars. Cloning and sequence analysis indicated that *flaB *is suitable in the detection of infection by pathogenic leptospires [93].

The virulence factor Hsp58 is a major target for the vaccine design [88]. The virulence factor hemolysin SphH is a pore-forming protein on several mammalian cells. The immune serum against the full-length hemolysin can effectively neutralize the SphH-mediated hemolytic and cytotoxic activities. SphH is required for pore formation in mammalian cell membranes and cytotoxic activities to mammalian cells [89,90]. The virulence factor ChpK is encoded by *L. interrogans chp *locus, which consists of two genes: *chpK *and *chpI*. Expression of *chpK *in *Escherichia coli *results in inhibition of bacterial growth. Coexpression of *chpI *neutralizes ChpK toxicity. The *chp *locus was found in all representative pathogenic strains of *L. interrogans *[91]. All virulence factors described above (Hsp58, FlaA, FlaB, SphH and ChpK) can be considered as candidates for leptospirosis vaccines.

**Figure 3:**
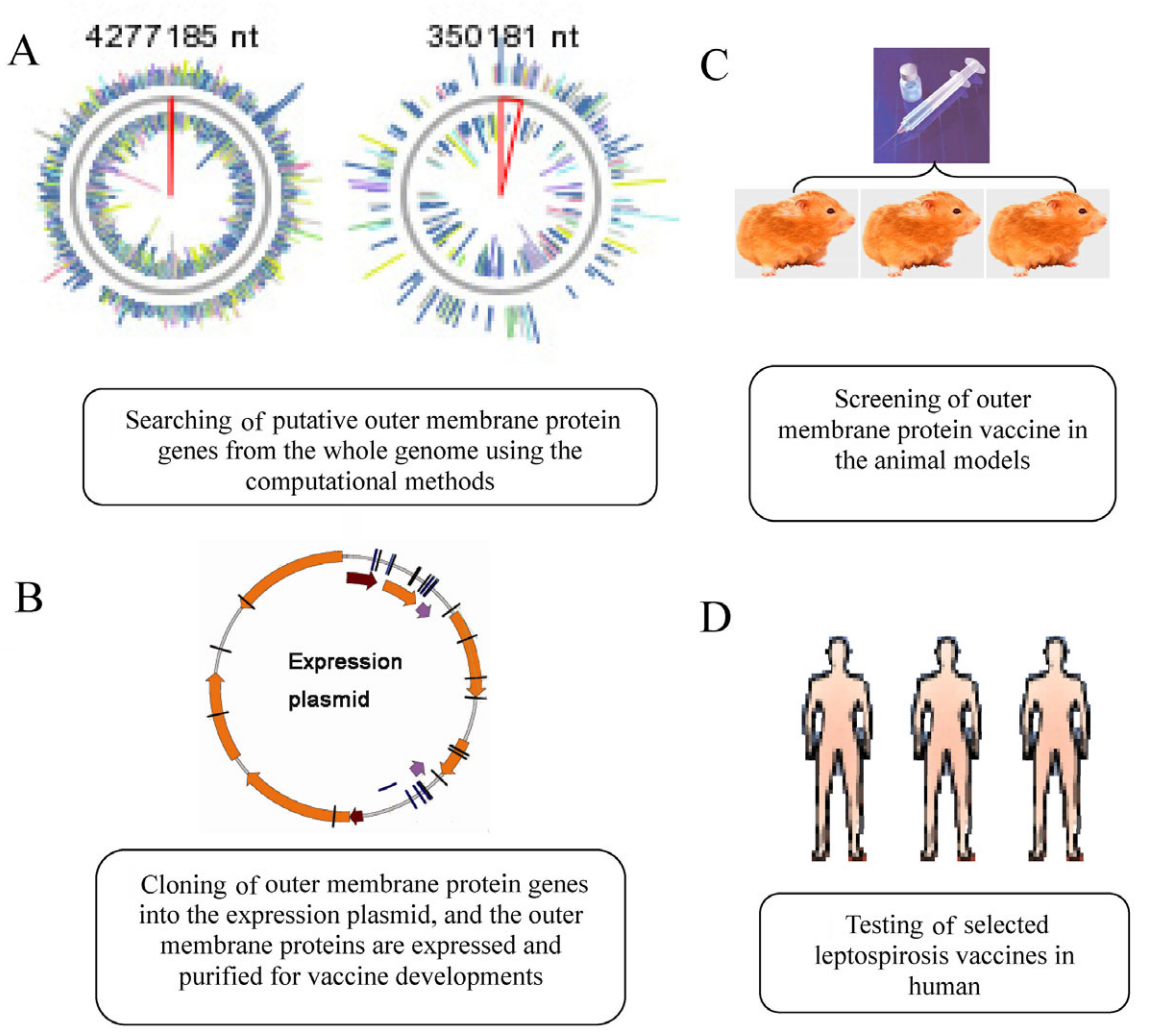
Large-scale screening of the leptospirosis vaccines. A): The screening experiment is started from the *Leptospira *genome sequence, B): Putative outer membrane protein genes are cloned into expression vector for recombinant protein production. C): Vaccination experiments are performed in an animal model. D): Selected vaccines are applied to human for clinical application testing.

Only Lig, LipL41 and Hap1 proteins were approved as vaccines against *Leptospira *in animals. Many recently reported outer membrane proteins (LAg42, Loa22, Lk73.5), lipoproteins (LipL32, LipL45, LipL21 and GLP) and newly discovered virulence factors (Hsp58, FlaA, FlaB, SphH and ChpK) can help us to find more suitable vaccine candidates [1-4,8,9,11-13,94]. Because the genomes of *L. interrogans *serovar Icterohaemorrhagiae Lai [63] and *L. interrogans *serovar Copenhageni [95] were reported, heterologous expression of leptospiral outer membrane proteins (OMPs) became possible and opened new possibilities for vaccine development [10]. The basic rout of large-scale screening of the leptospirosis vaccines is shown in Fig. 3. Before recombinant protein vaccines against leptospirosis can be used for clinical application, extensive testing is required. Recombinant protein vaccines must be free of contaminations, they should be stable and safe, and easy to transport and store.

## 3. LPS vaccines

Analysis of LPSs should open new avenues for vaccine developments [10,66]. The synthesis of LPSs in *Leptospira *is similar to that in other Gram-negative bacteria [42,96-98]. Leptospiral LPSs activate macrophages through CD14 and the Toll-like receptor 2 (TLR2) [40,99,100]. It is LPS, not lipoprotein, that stimulates the signaling component for macrophages through the TLR2 pathway [99]. Many reports have been published on leptospiral LPSs as vaccine candidates against leptospirosis [43-47,101-103].

It has been found that a LPS vaccine may be serovar-independent. For example, LPS vaccine prepared from *L. biflexa *serovar Patoc can effectively protect hamsters against *L. interrogans *serovar Manilae. In this case, the protective effect strongly depended on the dose and administration times of LPS vaccine prepared from *L. biflexa *serovar Patoc [104]. However, it was also reported that a LPS vaccine can be serovar-dependent; for example, LPS vaccines prepared from several different serovar strains could not induce a protective immune response in gerbils against the strains of different serovars [105]. Obviously, further studies are required to determine whether other LPS vaccines are serovar-dependent or -independent. If LPS vaccines are serovar-independent in different animals or human, it will make LPS vaccines more simple and efficient.

In 1989, a pioneer research on leptospiral LPS vaccines was reported [44]. Immunization characteristics of LPSs and polysaccharide (PS) in hamsters were compared. When hamsters were immunized with leptospiral LPSs or PS fraction from *L. interrogans *serovar Copenhageni, maximum titers were observed approximately 6 weeks after immunization. The protection was achieved by immunization with as little as 2.5 μg of LPS or PS [44]. The immune characteristics of LPS were compared with PS and immunoconjugate of PS and diphtheria toxoid (DT) in mice vaccinated with these compounds. The maximum agglutinin titers could be achieved at 6–10 weeks after vaccination with LPS or PS-DT conjugate. PS-DT gave antibody titers at least 10 times higher than those produced in response to LPS. Titers obtained in experiments with antigens of serovar Pomona were higher than those of serovar Hardjo [43]. The protective ability of LPS extracts were compared with the protein extracts in experiments in which leptospirosis was induced in gerbils [105]. Total extracts induced complete protection against homologous infections and partial protection against heterologous infections. LPS fractions only protected against homologous but not heterologous infections, whereas protein extracts caused a significant protection against both types of infections [105].

Moreover, an immunogenicity of oligosaccharide fraction from the LPS of *L. interrogans *serovar Pomona was reported [102]. The oligosaccharide was isolated by endo-glycosidase H digestion and column chromatography purification. When conjugated to diphtheria toxoid, the oligosaccharide caused production of a significant amount of protective antibodies [102].

Several lipopolysaccharide-like substances (LSSs) were reported as *Leptospira *antigens [47,106,107]. LSS was extracted from *L. interrogans *with a chloroform-methanol-water solution and partially purified by silica gel column chromatography. This antigen exhibited a protective activity in hamsters infected with lethal doses of *L. interrogans *[106,107]. Moreover, LLS extracted from *L. interrogans *by the hot phenol-water method could enhance the immunological response *in vivo *[47].

Since LPSs are major antigens involved in serological response [45], these molecules can be considered as candidates for serodiagnosis [41]. For example, although *L. borgpetersenii *subtype Hardjobovis and *L. interrogans *subtype Hardjoprajitno belong to different species, they are serologically indistinguishable, and thus classified as serovar Hardjo. This is because the LPSs of these subtypes are identical [103]. Hence, LPSs can be used not only as leptospirosis vaccines, but also as antigens for serodiagnostics.

Although LPSs have been tested as leptospirosis vaccines for almost 20 years, and promising results have been obtained, there are still have many problems to be solved. Between others, details of compositions and structures of leptospiral LPSs have to be determined.

## 4. Inactivated and attenuated vaccines

The inactivated and attenuated vaccines have been reported for more than 50 years. Some inactivated or attenuated leptospirosis vaccines were successfully tested in cattle [108-116] and dog [7,27,117-122]. Inactivated leptospirosis vaccines were also tested in human volunteers [12,123-126]. The sera from persons vaccinated with a bivalent whole cell inactivated vaccine of *L. interrogans *serovar Hardjo or serovar Pomona contained IgM specific to both serovars [125]. Although the only leptospirosis vaccine licensed for humans is being produced in Cuba since 2006, inactivated and attenuated vaccines still acquire considerable interests. They are especially suitable as veterinary vaccines. When dogs were vaccinated twice with such vaccines and infection with *L. interrogans *followed the second vaccination, a high rate of protection against *L. interrogans *was observed and duration of immunity was at least 1-year [127]. The efficiency of inactivated vaccine could be improved by adjuvant and vaccination frequency. A commercial inactivated leptospirosis vaccine (with adjuvant) induced a poor antibody production in cattle during preliminary vaccination. However, after booster vaccination, this vaccine caused a remarkable immune response [128]. Hydrostatic pressure-treated leptospires can be used as inactivated vaccine. After such leptospires were inoculated into rabbits, the vaccine was immunogenic [129]. The inactivated vaccine can induce a strong antigen-specific proliferative response by peripheral blood mononuclear cells (PBMC) of vaccinated cattle 2 months after the first dose of vaccine [130].

The results discussed above show that inactivated or attenuated vaccines are suitable as the leptospirosis vaccines [4,5,8-11,13]. However, such vaccines cause safety problems [131,132].

## 5. DNA vaccines

DNA vaccines have been used against different diseases [133]. These vaccines have several advantages over recombinant protein vaccines. Namely, DNA vaccines have very simple processing routes [134,135], low prices [136], and easy administration properties [137,138]. In just a few years, DNA vaccine technology has been developed from an interesting observation to the practical application [139-141]. Surprisingly, only two leptospiral DNA vaccine trials have been reported. DNA vaccine encoding hemolysis-associated protein 1 (Hap1) was tested in gerbils, and partial protection against the infection by pathogenic strains of *Leptospira *was achieved [142]. Another example is the use of a DNA vaccine containing the endoflagellin gene *flaB2 *in experiments with guinea pigs [143]. Obviously, leptospirosis DNA vaccines need more extensive investigations. It will be very valuable to test the immune characteristics of multiple leptospirosis DNA vaccine complexes in animals.

## 6. Leptospirosis vaccine ontology database

With the development of leptospirosis vaccines, it is now possible to construct a database for leptospirosis vaccines. We have constructed a leptospirosis vaccine ontology database, which is available at the website [69]. In this database, we have presented information on some important leptospirosis vaccines. Each vaccine in the database has an ontology value, obtained on the basis of the experimental results from particular report and expert evaluation. We believe that this database may be helpful for scientists working in the leptospirosis field, as well as for those working on bioinformatics. The information on vaccines can be transmitted to the bioinformatics field for vaccine classification, processing optimization and predicting of vaccine efficiency from amino acid sequences or compositions without experiments on animals.

## 7. Future developments in leptospirosis vaccine

Vaccines are administered to a large number of healthy humans and animals to make them resistant to diseases, therefore, vaccines must be of high safety [144,145]. There are two basic types of leptospirosis vaccines available, attenuated and inactivated leptospirosis vaccines. However, these two types of vaccines reveal significant safety problems.

The attenuated vaccines were achieved by propagation of the microbe under conditions different from those in the infected host and, hopefully, unfavorable to its growth in the host. Obviously, this method could not guarantee enough safety. It requires many tests to ensure the safety, but the safety still remains an issue. A more rational approach to attenuate the microbe would be to inactivate leptospiral components or genes known to code virulence factors. For example, an attenuated leptospiral vaccine could be developed by elimination of the leptospiral O antigen. Analogously, inactivation of the LPS biosynthetic loci (*rfb*) might result in attenuation a leptospiral strain. Careful analysis of genes coding for the virulence factors of *Leptospira *should allow us to construct attenuated strains for leptospirosis vaccines.

Inactivated leptospirosis vaccines were extensively investigated during 70–80s' of 20^th ^century. The major problems concerned both their safety and efficacy. It appears that a combination of attenuated and inactivated leptospirosis vaccine may be highly effective. New attenuated vaccines should be designed on the basis of our knowledge about sequences of genomes and virulence determinants of the pathogens to maximize their safety.

A real breakthrough for leptospirosis vaccines was the genetic technology that allows expression of leptospiral genes in heterologous organisms. In fact, many outer membrane proteins have been obtained, which are candidates for vaccines [58]. The advantages of production of recombinant vaccine antigens in a selected heterologous host organism arises from simplicities of cultivation of the host and purification of recombinant proteins. Furthermore, recombinant proteins are useful as antigens in immunoassays to detect leptospires.

It has been reported that leptospirosis vaccines with adjuvant were more immunogenic than those without adjuvant [146]. Thus, it appears that more research is required to develop novel adjuvants for leptospirosis vaccines, like recently described bacterial tRNA adjuvant [147] and cdiGMP adjuvant [140].

Increasing attention is being devoted to the fact that infections of leptospires are naturally acquired through mucosa. Administering the vaccines by the mucosal route [148-152], oral route [153-156], nasal spray [157], or through topical application on the skin would be welcome [138]. The optimal formulations, adjuvants, doses and schedules are crucial for vaccine efficacy [127,158]. The idea to produce leptospiral antigens as protein components of edible plants is indeed feasible, and the idea of the combination of leptospirosis vaccines and drugs to cure leptospirosis is very interesting [6,8,9,158]. An improvement of quality of DNA vaccines and recombinant protein vaccines appears to be important for the practical application [135,159-161]. Moreover, we believe that construction of a web service, like the leptospirosis vaccine ontology database, would be important for scientists working on leptospirosis vaccines.

## Competing interests

The author(s) declare that they have no competing interests.

## Authors' contributions

All authors have contributed to the content of the article.
